# Supplementary material for: MetaRibo-Seq measures translation in microbiomes
Source: Nat Commun. 2020 Jun 29;11:3268. doi: 10.1038/s41467-020-17081-z (PMC7324362; doi:10.1038/s41467-020-17081-z)
Supplement: Supplementary file 10 — Supplementary Data 7 [file 41467_2020_17081_MOESM10_ESM.zip › File2/Confidence_High_Taxonomy/179220_out.krona.html]

Javascript must be enabled to view this page.

members
magnitude
magnitudeUnassigned
count
unassigned
taxon
rank

179220\_out

39

2
39
superkingdom

39
phylum
1239

186801
39
class


SRS014683\_contig\_number\_contig-100\_25028.73257SRS015794\_contig\_number\_contig-100\_14955.14955SRS017521\_contig\_number\_contig-100\_12587.211228SRS019601\_contig\_number\_contig-100\_11510.117448SRS019910\_contig\_number\_contig-100\_24783.47946SRS043411\_contig\_number\_10337SRS098717\_contig\_number\_contig-100\_41542.41542SRS1041090\_contig\_number\_contig-100\_22390.22391SRS140492\_contig\_number\_9085
9
order
39
186802

216572
family
2

2
459786

SRS054590\_contig\_number\_1383
1
genus


SRS893253\_contig\_number\_2456
876091
species
1

541000
family
27

1924093
1
genus

1673717
1
species

SRS049402\_contig\_number\_8442

946234
genus
26

25
species
292800

SRS013098\_contig\_number\_20370SRS015217\_contig\_number\_11039SRS015264\_contig\_number\_5503SRS016132\_contig\_number\_4455SRS046712\_contig\_number\_4077SRS049402\_contig\_number\_6794SRS055966\_contig\_number\_1672SRS063127\_contig\_number\_2782SRS077454\_contig\_number\_6572SRS077502\_contig\_number\_11929SRS077730\_contig\_number\_contig-100\_4862.95719SRS098655\_contig\_number\_13062SRS1041095\_contig\_number\_contig-100\_3641.3642SRS1041147\_contig\_number\_contig-100\_3299.3300SRS143780\_contig\_number\_contig-100\_5852.163216SRS143876\_contig\_number\_9883SRS143991\_contig\_number\_15811SRS146764\_contig\_number\_30445SRS146888\_contig\_number\_contig-100\_2380.85381SRS147022\_contig\_number\_11855SRS147139\_contig\_number\_29710SRS148511\_contig\_number\_16252SRS148874\_contig\_number\_8226SRS149244\_contig\_number\_4021SRS893256\_contig\_number\_2015

species
1
1193534

SRS148511\_contig\_number\_5640

1
family
186803

658087
species
1

SRS017521\_contig\_number\_49775
